# Machine Learning-Assisted High-Throughput Screening for Electrocatalytic Hydrogen Evolution Reaction

**DOI:** 10.3390/molecules30040759

**Published:** 2025-02-07

**Authors:** Guohao Yin, Haiyan Zhu, Shanlin Chen, Tingting Li, Chou Wu, Shaobo Jia, Jianxiao Shang, Zhequn Ren, Tianhao Ding, Yawei Li

**Affiliations:** 1 Shaanxi Key Laboratory for Theoretical Physics Frontiers, Institute of Modern Physics, Northwest University, Xi’an 710069, China; y1144764721@163.com (G.Y.); chen18309275802@163.com (S.C.); 13571194917@163.com (T.L.); wuchou@stumail.nwu.edu.cn (C.W.); 202232630@stumail.nwu.edu.cn (S.J.); qazooo3212024@163.com (J.S.); renzhequn@yeah.net (Z.R.); 18392066590@163.com (T.D.); 2 Institute of Yulin Carbon Neutral College, Northwest University, Xi’an 719000, China; 3 School of Energy, Power and Mechanical Engineering, Institute of Energy and Power Innovation, North China Electric Power University, Beijing 102206, China

**Keywords:** hydrogen evolution reaction, machine learning, high throughput screening, density functional theory

## Abstract

Hydrogen as an environmentally friendly energy carrier, has many significant advantages, such as cleanliness, recyclability, and high calorific value of combustion, which makes it one of the major potential sources of energy supply in the future. Hydrogen evolution reaction (HER) is an important strategy to cope with the global energy shortage and environmental degradation, and given the large cost involved in HER, it is crucial to screen and develop stable and efficient catalysts. Compared with the traditional catalyst development model, the rapid development of data science and technology, especially machine learning technology, has shown great potential in the field of catalyst development in recent years. Among them, the research method of combining high-throughput computing and machine learning has received extensive attention in the field of materials science. Therefore, this paper provides a review of the recent research on combining high-throughput computing with machine learning to guide the development of HER electrocatalysts, covering the application of machine learning in constructing prediction models and extracting key features of catalytic activity. The future challenges and development directions of this field are also prospected, aiming to provide useful references and lessons for related research.

## 1. Introduction

Amid the worsening global energy crisis and environmental pollution, the pursuit of clean, sustainable, and efficient green energy has become a top priority [1]. Renewable energy sources such as wind, solar, and tidal energy have garnered significant attention and achieved remarkable progress. However, these energy sources face inherent limitations, particularly in terms of storage challenges and the instability of energy output in many scenarios. Consequently, identifying energy sources with superior performance has become an urgent endeavor [2]. Among these, hydrogen stands out as a promising energy carrier due to its environmentally friendly characteristics, including being clean, non-polluting, recyclable, and possessing a high calorific value, making it a key contender for future energy development.

**Figure 1:**
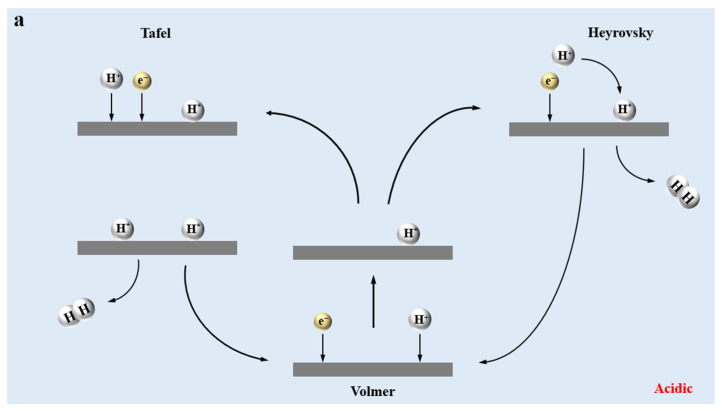

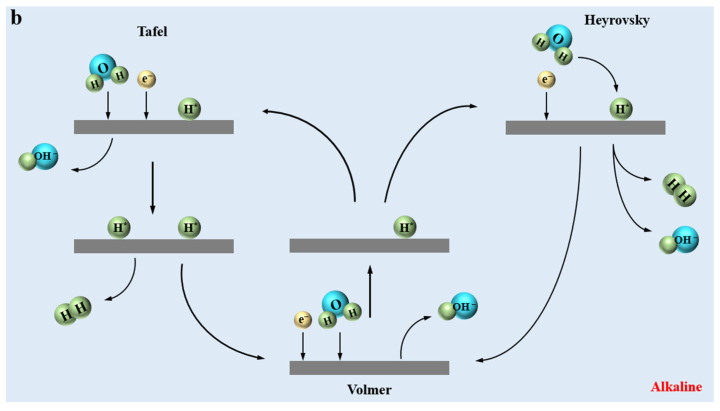
(**a**,**b**), respectively, represent the HER mechanisms in different reaction environments. The * represents the adsorbed state, and H* indicates hydrogen in the adsorbed state. Grey and green represent hydrogen from different sources, blue represents oxygen, and yellow represents electrons.

Hydrogen in nature primarily exists as compounds, commonly found in carbohydrates and water. Three hydrogen production methods have been developed to produce hydrogen efficiently and in an environmentally friendly and sustainable manner: methane reforming of water vapor, gasification of coal, and electrolysis of water. However, the first two ways of hydrogen production consume fossil fuels, which can lead to several environmental problems. In contrast, water electrolysis is regarded as a more promising and sustainable hydrogen production method [3]. HER, which is a critical step in the process of hydrogen production from water decomposition, plays an important role in clean energy conversion and has received much attention and research due to its sustainability, safety, and reliability. This electrocatalytic reaction, which occurs at the cathode, begins with the Volmer reaction, where protons in the solution adsorb onto the catalyst surface and combine with electrons to produce hydrogen atoms (H_ads_). The second step involves hydrogen generation through two possible pathways: (1) the Heyrovsky reaction, in which a new proton is trapped by the surface of the catalyst and combines with an electron and a H_ads_ to produce H_2_, or (2) the Tafel reaction, where two H_ads_ combine on the surface of the catalyst to produce H_2_. It is worth noting that in acidic and alkaline environments, the mechanisms of electrochemical hydrogen evolution reactions are slightly different. In acidic environments, the total reaction is as follows: 2H^+^ + 2e^−^ → H_2_, and the HER usually follows the Volmer–Heyrovsky and Volmer–Tafel pathways. In alkaline environments, where water molecules serve as the primary proton source due to the solvent’s lack of free protons, the Volmer reaction reduces water molecules adsorbed on the catalyst, producing H_ads_ and OH. Subsequently, in the Heyrovsky reaction, water molecules combine with electrons and H_ads_ to produce H_2_ and OH^−^. The Tafel reaction in alkaline environments remains consistent with acidic conditions, involving the combination of two H_ads_ to produce H_2_. The overall reaction of the HER is as follows: 2H_2_O + 2e^−^ → H_2_ + 2OH^−^, following the Volmer–Heyrovsky reaction corresponding to the alkaline environment and the Volmer–Tafel reaction, as shown in Figure 1. Despite its potential, HER typically requires a high onset potential and has a low hydrogen production rate, leading to significant energy consumption [4]. Consequently, the development of efficient HER electrocatalysts has become the core of the achieving of hydrogen production from electrolytic water. Among them, Pt-based catalysts have received much attention due to their excellent catalytic performance and good stability [5,6,7]. However, their high economic cost has greatly restricted their application in the industrial field. As a result, the economic feasibility of HER electrocatalysts has become a critical consideration in their development.

To reduce the overpotential of HER, the development of high-performance, low-cost HER electrocatalysts has emerged as a key focus in energy research. However, the relationship between the electronic structure of electrocatalysts and their catalytic performance is highly complex. Traditional approaches to HER catalyst development often rely on the trial-and-error method [8,9], which consumes substantial time, manpower, and material resources. This approach limits the ability to address fundamental issues and hinders the implementation of innovative designs. Although high-throughput computing can process large datasets in a short time and provide valuable insights into electrocatalyst research, challenges remain in efficiently analyzing and extracting critical information from extensive datasets.

The advent of machine learning models offers new strategies to address these challenges. Machine learning, a key component of data science and technology, excels in handling complex, high-dimensional datasets containing multiple variables. Its powerful capabilities for data analysis and prediction allow it to extract valuable insights and uncover hidden information from massive datasets. Furthermore, the integration of machine learning and high-throughput screening can establish the constitutive relationship between the microstructure and catalytic performance of materials. This integration enables the rapid identification of potential candidates with excellent HER catalytic performance. For example, Mao et al. investigated 7924 Cu-based alloy candidates and identified NiCu alloy clusters as excellent HER electrocatalysts. Their study demonstrated that charge transfer around the adsorption sites could be an excellent descriptor related to the free energy of HER by applying machine learning to search for the descriptors [10]. With the ongoing advancements in high-throughput computing, machine learning techniques, and new material exploration, machine learning shows great potential in HER catalyst screening. However, challenges persist, including data quality and quantity, model interpretability, and the ability to perform multiscale simulations. Through continuous innovation and integration, these challenges can be addressed, driving significant progress in catalyst design and contributing to the advancement of hydrogen energy technology.

This paper provides a review of the relevant research on high-throughput computing combined with machine learning to guide catalyst development in recent years, covering the basic principles of density functional theory, the application of machine learning in predicting novel catalysts, and mining catalytic activity-related features. The aim is to review the research progress of machine learning-based high-throughput screening of electrocatalysts for the hydrogen evolution reaction, which will provide useful references and lessons for subsequent related studies. Finally, challenges and perspectives of machine learning in HER research are presented.

## 2. High-Throughput Screening and Machine Learning

### 2.1. High-Throughput Screening of HER Catalysts

In electrocatalytic HER, high-throughput calculations can simultaneously test the activity, selectivity, and stability in the chemical reactions of hundreds or even thousands of catalysts with different compositions or structures, increasing the probability of discovering new and efficient catalysts, and decreasing the time and cost of finding high-performance catalysts with the traditional trial-and-error method. Density Functional Theory (DFT)-based computational methods and processes can be integrated into the framework of high-throughput computation by writing scripts or using specialized computational software to automate computation and data processing for multiple systems, thus improving computational efficiency and rapidly obtaining a large amount of valuable information.

In 1964, Hohenberg and Kohn [11] introduced the DFT, proposing that the ground-state energy of materials could be expressed solely through functionals of the electron density, this groundbreaking work laid the foundation for DFT. The following year, they presented the Kohn–Sham equations [12,13], which have since become the preeminent approach for handling DFT. This approach enables the description of complex interactions among electrons and between electrons and atomic nuclei in terms of electron density. During the solution process, to mitigate computational complexity, suitable approximation methods are essential. The local density approximation (LDA) [14] represents the most straightforward approximation method, while the generalized gradient approximation (GGA) [15] is more prevalently employed in contemporary research. In materials science, common GGA functionals include Perdew–Wang91 (PW91) [16] and Perdew–Burke–Ernzerhof (PBE) [15]. However, as a semi-local functional, the GGA functional cannot accurately describe long-range interactions. Thus, the Grimme method can be employed for the dispersion correction of long-range van der Waals interactions.

In catalysis research, catalytic activity descriptors are physical or chemical quantities that quantitatively or qualitatively describe the activity of a catalyst. These descriptors are closely related to the catalyst activity, for example, the Gibbs free energy change in adsorbed hydrogen (ΔG_H*_) is represented by the formation of H*, which can be calculated as follows:

ΔG_H*_ = Δ*E_ads_* + Δ*E_ZPE_* + *T*Δ*S*, (1)

where Δ*E_ads_* and Δ*E_ZPE_* indicate the adsorption energy and zero-point energy correction based on DFT calculations. *T* and Δ*S* represent the temperature and the entropy change. In the volcano–model relationship [17] established between the experimentally measured exchange current density *i*_0_ of catalytic materials and ΔG_H*_, the reaction attains its peak rate when ΔG_H*_ nears zero. Specifically, when ΔG_H*_ ≈ 0 [18,19], the catalyst exhibits superior catalytic activity. However, although ΔG_H*_ provides a quantitative theoretical basis for the activity criterion of HER catalysts, it cannot predict potential new catalysts [20]. Thus, there is a pressing need to develop other catalytic descriptors capable of uncovering the reaction mechanism. With the application of DFT in the catalytic domain, leveraging catalytic activity descriptors as a bridge between high throughput screening and DFT has opened up the possibility of constructing completely new catalytic activity descriptors.

Overall, the calculation process of DFT is a relatively complex mathematical treatment. In practical research, the VASP (Version 6.5.0) (Vienna Ab initio Simulation Package) [21] is commonly employed to compute various material properties. To perform high-throughput calculations with VASP efficiently, supplementary software is often required. For instance, VASPKIT (Version 1.5.0) [22] can handle both the pre- and post-processing steps of VASP calculations through an integrated interface.

### 2.2. Overview of Machine Learning

Machine learning, a critical branch within the field of Artificial Intelligence (AI) [23], enables the construction of model assumptions about the subject of study through the input of relevant data, iterative optimization of the model, and subsequent achievement of data-driven decision-making or prediction. Currently, researchers are using an increasingly rich set of machine learning models to advance research in materials science.

Based on the learning approaches, machine learning models are categorized into two primary types: supervised and unsupervised learning. Both types have unique advantages in descriptor searches and catalyst discovery, operating both independently and synergistically. Furthermore, semi-supervised learning combines the features of supervised and unsupervised learning. In the process of machine learning, the effect of different model training in different systems is different, and it is especially important to choose the right model. The development of machine learning has produced a rich variety of models. Currently, widely used classical machine learning models include the following: Multiple Linear Regression, Support Vector Machines, Decision Trees, Random Forests, Extreme Gradient Boosting, and so on.

Multiple Linear Regression (MLR) is one of the simplest machine learning models [24]. In the field of catalysis, the linear regression model can quickly find the source of HER catalytic performance, but the fitting effect is poor for complex nonlinear problems. In this regard, the Kernel-based learning model can reduce the disturbing factors of the input data and thus obtain an accurate linear relationship between the input and output data [25].

Support Vector Machine (SVM) [26] is a classical supervised learning model. It classifies by partitioning diverse training data, all the while maintaining a minor error margin. When data defies easy separation, introducing a kernel function renders it linearly separable [27]. The model is suitable for some small samples, nonlinear, high-dimensional data and other cases of more datasets [28], but there is a certain limitation on the number of samples, and its computational complexity rises with the increase in the number of samples.

Decision Tree (DT) [29] partitions datasets according to feature importance, constructing a tree-like structure for classification or regression. Each internal node denotes a feature test, each branch represents a possible feature value, and each leaf node represents the final decision category or predicted value. It is suitable for handling data with explicit feature attributes that can be predicted by classification or continuous values.

Random Forest models (RF) [30] are based on decision trees and thus constructed, and can be considered as a combination of a series of DT models with high accuracy and good stability [31]. During the construction of each DT model, multiple different training sets are constructed by sampling the initial training data with putback, which increases the diversity of the models.

Extreme Gradient Boosting (XGB) [32], originating from an open-source program project, relies on decision trees in its algorithm. Its core concept involves generating a new weak learner (typically a decision tree) in each iteration to fit the residuals between the predictions of the previous learner and the target values. As more learners are added iteratively, it converges towards the target value. The final regression value is derived by summing the outputs of all weak learners. Thanks to its outstanding numerical-fitting capabilities, XGB finds extensive use in computational materials science.

In addition to the above machine learning models, numerous other machine learning models have either been extensively applied or are still in development. In the context of HER catalyst exploration, these emerging models continue to drive breakthroughs and discoveries in the field of catalysis. In summary, machine learning can predict the exact descriptors or new materials for HER by collecting computationally independent data, and Table 1 summarizes the advantages, disadvantages, and scope of application of the above classical machine learning models, which facilitates the direct selection of the corresponding models.

### 2.3. Machine Learning Combined with High-Throughput Screening

HER electrocatalysts play a crucial role in electrochemical and energy conversion technologies by improving the efficiency of converting water to hydrogen. The development of suitable catalysts is the key to achieving hydroelectrical hydrogen resolution. However, due to the complexity of electrocatalytic systems and the multiplicity of candidate catalyst materials, the exploration and development of HER electrocatalysts face a dilemma of how to break away from the constant inefficient trial-and-error model and develop efficient and rational research and development models. Currently, the emergence of machine learning tools offers new opportunities for addressing these challenges in materials research.

The main applications of machine learning in the field of HER catalysis are divided into two areas. Firstly, machine learning can assist in material screening. By leveraging limited datasets, machine learning models can establish relationships between input and output data, enabling the accurate prediction of novel materials and their properties.

In the performance evaluation of HER catalysts, according to Sabatier’s principle, the adsorption intermediates should exhibit moderate adsorption energy during the screening of catalysts. Weak adsorption hampers the adsorption of reactants, making the Volmer process unfavorable; while overly strong adsorption hinders the detachment of products, negatively impacting the Tafel or Heyrovsky process. It is worth noting that the electronic nature of the catalyst itself can have a great impact on the HER catalytic efficiency, and the ideal electrocatalyst should have appropriate H adsorption capacity in acidic environment, i.e., the catalyst can exhibit excellent HER catalytic performance when ΔG_H*_ is close to zero. However, in alkaline environments, the HER becomes more complex due to the altered reaction mechanism, which makes it more difficult to find a reasonable descriptor to explain the catalytic activity. By leveraging machine learning, datasets can be used to construct models that correlate material properties, enabling the identification of optimal descriptors for catalytic performance. This facilitates the rapid screening of candidate materials with desired properties. For example, Tran et al. [33] developed a screening framework to guide theoretical calculations through a machine learning model that accurately searched a large space and ultimately screened 131 CO_2_RR and 258 HER catalysts, respectively.

In addition, machine learning can assist in mechanistic analyses, and understanding catalytic reaction mechanisms through machine learning models is often more important than the models themselves. Machine learning, as a powerful data processing and analysis tool capable of handling complex datasets, offers new ways to explain catalytic reaction mechanisms. Han [34] used SISSO [35], which combines mathematical operators with input independent variables to establish an explicit mapping relationship between inputs and target quantities. They proposed a new descriptor for the hydrogen adsorption stability of single-atom catalysts, revealing the co-dependence of H stability on the adsorption free energy and energy barrier.

**Figure 2:**
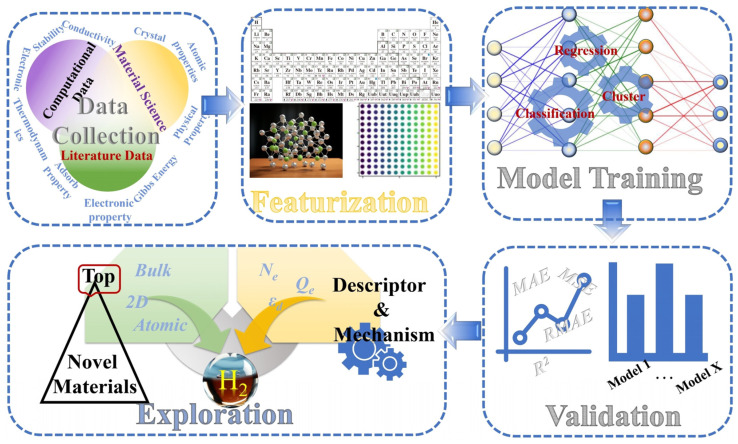
The schematic diagram for the workflow of the machine learning in HER electrocatalysts exploration. Reproduced from Ref. [36] with permission.

Furthermore, systematic workflows for applying machine learning in electrocatalytic HER research have been proposed. For example, Zhang et al. [36] outlined a general workflow comprising key steps such as data collection and characterization, model training as well as validation, and finally exploring the unknown material space by using machine learning models. as shown in Figure 2.

In general, a complete catalyst exploration study for machine learning engagement often includes the following phases: Firstly, in the data collection and database construction phase, obtaining accurate electrocatalysis-related data from diverse sources is essential for ensuring the accuracy and reliability of the final model. Undoubtedly, data collection is the linchpin of machine learning [37]. From the initial data gathering to the establishment of a comprehensive training model database, the process encompasses complex steps like data screening and integration, which are both cumbersome and highly challenging. Researchers have made substantial contributions to the systematic collection, organization of data, and database construction.

Typically, training data for machine learning primarily derives from material science databases, theoretical calculation databases, and literature databases. Under varying experimental conditions, researchers conduct performance tests on diverse electrocatalytic materials for the hydrogen evolution reaction. They collect crucial data such as overpotential, Tafel slope, current density, and stability. Moreover, they utilize advanced characterization techniques to analyze the microstructure in detail, thereby obtaining information on the crystal structure characteristics and electronic structure of the materials. Over a long-term development, material science databases, including the Crystallography Open Database and the Inorganic Crystal Structure Database, have been gradually established. These databases can supply a vast amount of reliable raw data for machine learning. Currently, although these datasets are still evolving and continuously updating a large volume of data verified by theory and experiments, they are insufficient to address all material systems. For special systems, computational methods like DFT are still required to acquire data on the electronic structure and adsorption energy of catalytic materials. Notably, the raw data from theoretical calculations may contain errors. Thus, prior to collecting DFT data, it is imperative to clarify the electrocatalytic problems to be solved and validate them with experimental results to guarantee the accuracy of the theoretical data. Furthermore, existing literature databases, founded on the theories or experiments of complex systems, can significantly enhance the overall dataset quality. Through a systematic analysis of published relevant research literature, researchers can extract information about the performance data and material structures of electrocatalytic materials for the hydrogen evolution reaction and select representative and reliable data for inclusion in the training database.

After data collection, the collected data must be processed to eliminate noise, duplicate data, and incorrect data. A specialized data storage and management system should be developed to ensure the safe, accurate, and efficient storage of data. Appropriate data formats and database architectures should be adopted to enable rapid data entry, querying, and updating. Simultaneously, a data backup and recovery mechanism should be established to prevent data loss.

The second stage is the feature engineering stage, in general, in HER research, features are typically divided into two categories. The first category relates to the origins of catalytic activity and includes parameters such as Gibbs free energy, adsorption energy, geometrical features (e.g., bond lengths of adsorbed hydrogen, bond angles, and the local environment of the active site), d-band centers, charge transfer, electronegativity, and other electronic features relevant to proton-electron transfer. The second category focuses on identifying potential catalyst features, including geometric and electronic properties like crystal structure, stability, and conductivity. This stage involves extracting features from raw data that reflect electrocatalytic properties, such as physicochemical attributes, structural parameters, and reaction conditions. For example, the elemental composition, structural information, and electronic properties of the catalyst. Then, from the many features, the features with high relevance to the prediction target and significant effect on the model performance improvement are filtered out, and the redundant and irrelevant features are removed to reduce the complexity of the model and improve the generalization ability of the model. This process can be carried out by using correlation analysis, principal component analysis and other methods for feature selection, e.g., Chen et al. [38] used a Pearson correlation coefficient matrix and feature importance assessment to select four critical features for training a Gradient Boosting Regression (GBR) model. Following feature selection, preprocessing techniques like normalization, standardization, or logarithmic transformation are applied to ensure numerical stability and comparability, enhancing model training efficiency and convergence speed.

The third stage is the model training stage, where suitable machine learning models, such as linear regression, decision trees, or support vector machines (SVM), are chosen based on the problem’s nature and the data’s characteristics. The processed dataset is typically split into training and test sets, often in an 80:20 or 70:30 ratio. The selected model is trained using the training set, and by adjusting the parameters of the model, the model is able to learn the laws and patterns in the data, so that the prediction error is minimized.

The fourth stage is the model validation stage, when the training is completed, the model can be validated by cross-validation methods to ensure its prediction accuracy and generalization ability. Commonly used cross-validation methods include k-fold cross-validation [39], leave-P-out-cross-validation [40] and Bootstrap cross-validation [41]. In electrocatalytic HER, models can be selected by evaluating the mean absolute error (MAE), mean square error (MSE), and root mean square error (RMSE), smaller values indicate a smaller overall model error and more accurate prediction results. Comparing the results of different models under various metrics can improve the accuracy of the final model selection. The results of the DFT calculations are often used during the research process to compare and analyze the predictions of the models, to further validate the predictions and compare the models, and if the models do not perform well, try adjusting the parameters of the models, selecting different features, or even trying different types of machine learning models.

Finally, there is the model application stage, where the models validated through evaluation are used to apply to real electrocatalytic problems to perform tasks such as prediction, classification, and optimization. For example, predicting the performance of unknown electrocatalysts and selecting candidate catalysts with good catalytic performance. In fact, although the machine learning model can provide predictions, the final selected candidate catalyst still needs to undergo actual catalytic experiments to verify its performance. Based on the experimental results, the model can be further optimized or the next round of prediction and experiments can be conducted.

In the development process of the integration of electrocatalysis and machine learning, to meet the needs of researchers and engineers in electrocatalysis research and applications, various mainstream electrocatalysis machine learning software have emerged. The software typically integrates a wealth of machine learning algorithms and tools, and offers user-friendly interfaces and convenient data processing functions, enabling users to easily conduct model training, prediction, and analysis. For example, Scikit-learn, a mainstream open-source machine learning toolkit, provides a rich set of traditional machine learning algorithms. These algorithms are simple and fast, and can be used for the analysis and modeling of electrocatalytic data. Scikit-learn has a simple API and interaction logic, and supports functions such as data pre-processing, model selection, and evaluation, making it suitable for beginners and rapid experiments. In addition, Pytorch is an open-source optimized tensor library for building deep machine learning models. Thanks to its characteristic of dynamic computational graphs, it greatly facilitates researchers in debugging and modifying models. Moreover, its code style is concise and easy to understand, making it suitable for quickly building and iterating models. Relevant technologies and resources can provide reference for electrocatalysis research.

## 3. Machine Learning-Assisted High-Throughput Screening

The study of new materials is often a time-consuming and complex process. High-throughput computation can provide more accurate results, but as the structure of materials becomes more diverse and complex, high-throughput computation of large systems or complex systems relying only on DFT encounters many serious challenges, which greatly limits the exploration of HER electrocatalysts. Machine learning is a crucial modern technology that, when combined with high-throughput computation, is able to construct predictive models that are more efficient and accurate than DFT computation [42], thus enabling rapid screening of catalysts and discovery of novel descriptors. In recent years, machine learning has been widely used as an efficient aid in computational materials science research and has been reported in several fields [43,44,45]. In this part, the specific application of machine learning combined with high-throughput computing in the screening process of various types of HER electrocatalysts will be explored in depth.

### 3.1. Noble/Non-Noble Metal Electrocatalysts

After decades of exploration, platinum-based metal catalysts are widely used in commercial applications due to their excellent catalytic properties [46]. Studies on various metals have shown that the ΔG_H*_ of metals have a volcano shape, with the top of the volcano representing the best performance of the metal in HER. Among these metallic materials, platinum is at the top of the volcano figure, so platinum is considered to be the most suitable catalyst material. Based on this, researchers have given priority to applying machine learning technology in optimizing platinum, platinum alloys, and more complex compositions.

**Figure 3:**
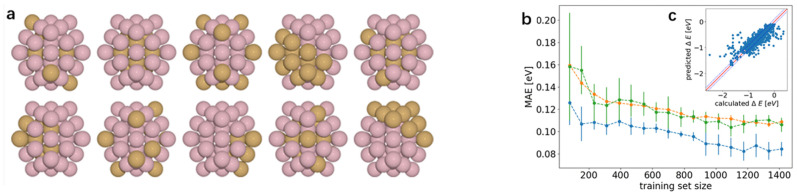
(**a**) Clusters of a given composition (e.g., the depicted Cu_13_Co_42_) were generated automatically by Monte Carlo assuming various combinations of interaction and segregation energies. Experimentally observable composites such as core–shell, segregated, ordered, and random as well as structures in-between emerged naturally. (**b**) Learning curve of KRR. The errors are averaged over 20 randomized runs and the error bars indicate the standard deviation of those errors. Training, validation and test set are in blue, yellow, and green, respectively. (**c**) Calculated vs. predicted hydrogen adsorption energy of 1767 DFT calculations. The deviation of data points from the diagonal directly indicates the disparity between predicted and calculated values, thereby assessing the model’s adsorption energy prediction accuracy. Reproduced from Ref. [48] with permission.

Currently, one of the methods that can effectively improve the catalytic performance of pure metal is to design polymetallic alloys containing optimal HER electrocatalyst compositions Pt. Based on this, the machine learning model is widely regarded as a powerful tool for screening HER electrocatalyst design. For example, Li et al. [47] mainly investigated the catalytic performance of transition metal alloy surfaces with strong (Pd, Pt) and weak metals (Ag, Au, Cu). DFT calculations and machine learning models were utilized to provide guidance for the design of HER catalysts. For 270 bimetallic alloys with different structures, they collected more than 450 hydrogen binding energies as well as 26 physical features such as electronegativity, d-orbital information, and d-band centers as input features. Ultimately, Pd_4_Ag_1−x_ and Pd_4_Au_1−x_ (100) possessed potential HER theoretical activities in acidic media and were screened as potential HER electrocatalyst candidates. This study fills the gap in the study of alloying effects on the (100) surface, especially the exploration of HER catalyst design on the atomic cluster scale, which provides a new research direction in this field. Similarly, Jäger et al. [48] focused on a different model system, which is a bimetallic icosahedral platinum nanocluster consisting of 55 atoms, which are binary assembled from the elements titanium, iron, cobalt, nickel, and copper. Their strategy in input feature engineering is to combine descriptors obtained based on smoothed overlap of atomic positions (SOAP) with the local density of states. And the Kernel Ridge (KR) regressor is applied as a machine learning model, supplemented with additional training sets during the cycle. After 1767 DFT calculations, it was able to achieve a mean absolute error (MAE) of 0.1 eV, as shown in Figure 3. Ultimately, the researchers discovered the advantages of Ni not only in binary alloys, but also nickel–cobalt (NiCo) and nickel–titanium (NiTi) in ternary platinum alloys. These studies show that machine learning combined with DFT can effectively facilitate the discovery of HER electrocatalysts, thus clearing the way for screening HER electrocatalysts with excellent catalytic performance.

**Figure 4:**
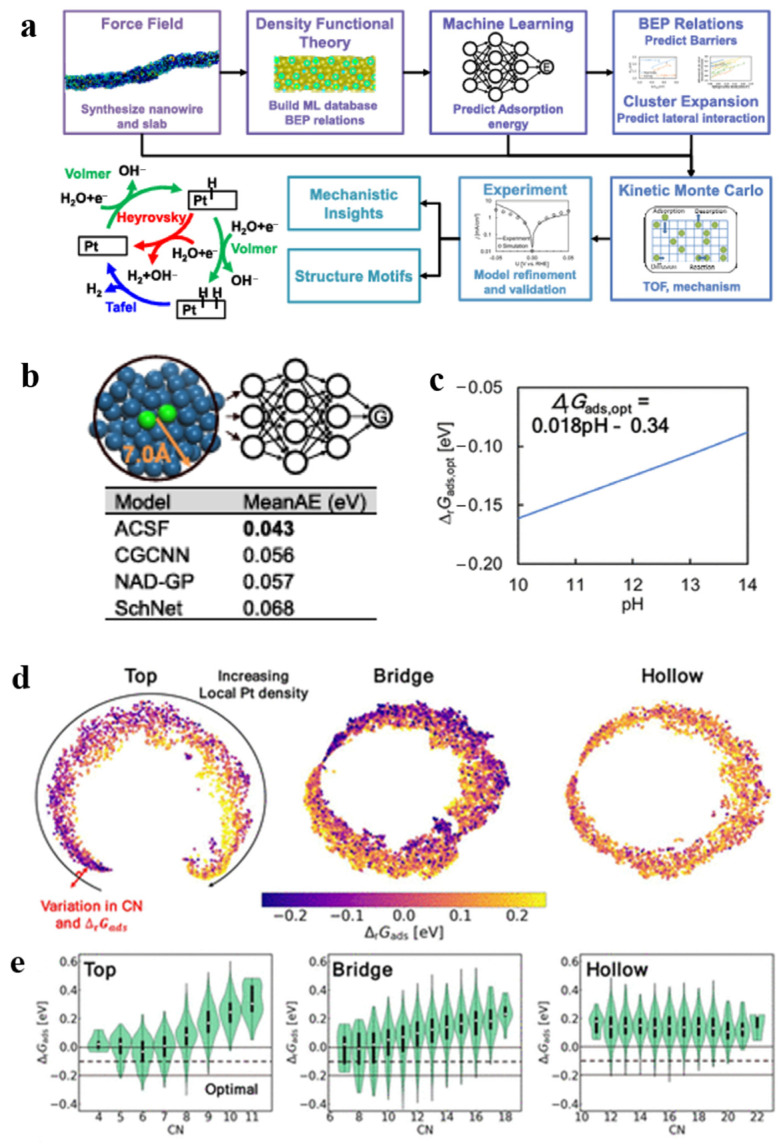
(**a**) End-to-end workflow for the jagged Pt nanowire simulation. (**b**) Δ_r_*G_ads_* prediction machine-learning architecture and the mean absolute error (MeanAE) for the tested models. (**c**) Optimal Gibbs free energy of adsorption, Δ_r_*G_ads,opt_*, vs. the pH at 0 V vs. RHE. (**d**) Points represent the sites and the distances between them, signifying the dissimilarity among sites, are visualized through the use of a SOAP descriptor, an average kernel, and t-SNE dimensional reduction analysis. (**e**) The Δ*_r_G_ads_* values are plotted against the coordination numbers. Reproduced from Ref. [50] with permission.

Machine learning modeling is not only useful for selecting excellent electrocatalysts, but also a powerful tool for deepening the understanding of electrocatalysts and grasping the design rules at the level of reaction mechanism. Ooka et al. [49] investigated the hydrogen binding energy on the surface of the catalyst platinum (Pt), which is different from the conventional thermo-neutrality study, and this study has brought about a significant shift in the deeper understanding of the design rules of HER. They derived their data from experimentally obtained electrochemical data and used a combination of regression modeling and genetic algorithms (GA) to estimate the binding energy more accurately. Their results show that the binding energy of hydrogen on the polycrystalline platinum surface is ΔG_H*_ = 0.094 ± 0.002 eV. It is important to consider overpotentials during catalyst design and that non-thermal neutral binding energies may be required to achieve optimal catalytic efficiency under conditions that deviate from equilibrium. This finding opens up new directions for the design of more efficient electrocatalysts for hydrogen precipitation reactions. In another study, Gu et al. [50] provided a new perspective to understand the complex kinetic phenomena on the nanoscale by using their machine learning approach to simulate the HER mechanism of serrated Pt nanowires under alkaline conditions (Figure 4a). They collected local environmental data from 3413 binding sites on serrated Pt nanowires as input features for training a machine learning model. The ACSF model was ultimately identified as the best-performing model due to its lowest error, with an average absolute error of only 0.043 eV (Figure 4b). The results showed that the optimal ΔG_H*_ values varied under different reaction conditions (Figure 4c) and that the sawtooth Pt nanowires possessed a self-dual functional mechanism, i.e., protons were adsorbed at the strong binding sites while hydrogen was activated at the weak binding sites. In addition, the machine learning simulation results also show that ΔG_H*_ is correlated with the coordination number (CN) (Figure 4d), specifically, the exposed sites with lower coordination numbers are closer to the optimal ΔG_H*_ value. Previously, such results, which require extensive simulations for statistical analysis, are now possible with DFT Machine Learning, which also facilitates a deeper understanding of complex dynamical processes and HER mechanisms at the nanoscale.

Traditionally, Pt-based and its alloy catalysts have been considered as the best HER catalyst candidates due to their excellent catalytic efficiency. However, research on catalysts mainly focuses on two objectives: improving the catalytic efficiency of HER catalysts themselves and reducing the demand for precious metal materials. In view of this, research on non-precious metal-based catalysts has developed tremendously. For example, Hoyt et al. [51] carried out an in-depth investigation of the ΔG_H*_ value on the surface of Ag alloy (211). Using DFT calculations, they obtained data on the adsorption energy of H on the surface of Ag (211) alloy, with a total of 5457 data points, and used this to construct a dataset for training a number of different machine learning models. After a thorough comparison and rigorous validation process, the researchers ultimately selected the Random Forest (RF) model as the top-performing model, which had a median absolute test error of only 14 meV. It was shown that the model, in addition to being able to make accurate predictions, revealed a range of chemical trends regarding H adsorption as well as counterintuitive behavior. For example, the relationship between adsorption energy and the position of doped atoms, and the non-monotonic change in adsorption energy in the dual-atom doped structure. This research further highlights the great application potential of machine learning in the field of electrocatalyst research, and also provides the important theoretical basis and practical guidance for subsequent related research.

**Figure 5:**
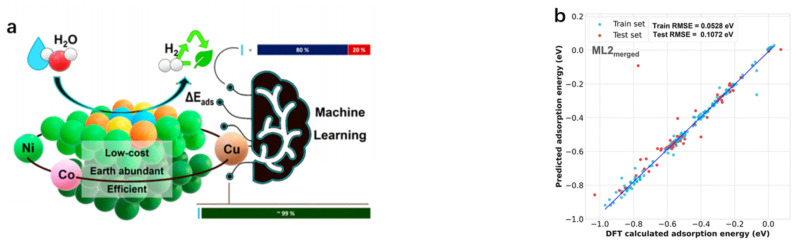
(**a**) Rational designing of bimetallic/trimetallic hydrogen evolution reaction catalysts using supervised machine learning. (**b**) Plot of DFT calculated adsorption energies (Δ*E_calc_*) versus predicted adsorption energies (Δ*E_pred_*) with its indicated Test and Train RMSE values for merged four datasets with optimized XGBR model. ML2 represents method 2 by considering merged datasets. Reproduced from Ref. [55] with permission.

As machine learning is deeply studied in catalyst development, its application faces the difficulty of balancing efficiency and accuracy, which mainly stems from two major problems. On the one hand, the computational cost of obtaining high-precision electronic or geometrical structures is high; on the other hand, those easy-to-obtain and relatively simple physicochemical properties can have a high error due to their low accuracy [52,53]. In this regard, Chen et al. [54] proposed an innovative machine learning framework, which provide the possibility of screening HER electrocatalyst alloys quickly and accurately. With this framework, they successfully screened 43 high-performance alloys from as many as 2973 candidate alloys that are expected to be potential electrocatalysts for HER, and some of the best candidates identified from this framework have been validated in experiments. Particularly importantly, this approach not only fully utilizes the potential of machine learning for efficient structural description, but also takes into account the consideration of simple physical properties. This undoubtedly suggests that the machine learning model has the potential to be further extended by effectively narrowing down the candidate space consisting of a large number of different combinations of metal elements, thereby accelerating the process of determining the optimal structure. Pandit et al. [55] conducted an in-depth study of non-Pt-based catalysts, including Cu, Co, and Ni in pure metal, bimetallic, and trimetallic alloys (Figure 5). They combined DFT with supervised machine learning techniques to design microstructural models, and an optimized extreme gradient boosting regression (XGBR) model was used to screen 27 promising and cost-effective non-Pt-based HER electrocatalysts from 5400 candidate systems, providing the basis for the development of highly efficient and cost-effective HER electrocatalysts. Which provides a new way to develop efficient and cost-effective electrocatalysts. Overall, the high-throughput screening and machine learning-based approach in this section can effectively accelerate the design of HER catalysts containing single to multi-metallic elements, while further discussing the HER mechanism therein as well as the potential factors affecting the catalytic activity. However, due to the very large experimental potential structure of HER, relevant studies are still scarce, and thus special attention should be paid to this area in future studies.

### 3.2. Single/Dual Atom Electrocatalysts

With the in-depth study of multiphase catalysis, the HER electrocatalytic efficiency can be significantly improved by dispersing single-atom catalysts (SACs) obtained by dispersing the active atoms with low loading rate on suitable substrates, and SACs have attracted wide attention due to their own unique properties and extremely high atom utilization efficiency [56,57,58]. DFT is widely used in the theoretical design of single-atom catalysts with high activity, stability, and selectivity. However, SACs have a wide range of candidate atoms and numerous substrate choices, and their complex structure–catalytic activity relationships require a large number of DFT calculations in a large parameter space, which entails significant time and computational costs. Machine learning, as a fast, accurate and low-cost auxiliary tool, can not only effectively reduce the pressure on limited computational resources, but also be used to predict the properties of SACs for their rational design [34,59,60].

The combination of machine learning and DFT calculations opens up new directions for the rapid and cost-effective rational design of SACs predicted to have optimal HER catalytic activity. With machine learning, existing datasets from DFT calculations can be used to construct databases that can be used for in-depth analyses and the establishment of structure–activity relationships, thereby accelerating the screening of SACs and reducing computational time and cost [61]. For example, Sun et al. [62] combined machine learning models with DFT calculations to screen and design HER catalysts in single metal atom doped Mbenes. Through model prediction comparisons, error analyses, and performance validation, the support vector regression (SVR) model emerged as the best choice due to its simplicity, efficiency, and stable prediction capabilities. Their study identified Bader charge transfer as a key factor influencing HER activity in Mxenes and Mbenes. Finally, they initially screened 28 candidate systems from 271 Mbenes and Mxenes systems by DFT calculation combined with the SVR model. After the second screening, stable Co_2_B_2_ and Mn/Co_2_B_2_ were identified as efficient HER catalysts as their |ΔG_H*_| were all less than 0.5 eV. Similarly, based on DFT calculations of 104 graphene-loaded single-atom catalysts (M@C_3_, M@C_4_, M@pyridinr-N_4_, and M@pyrrole-N_4_), Lin et al. [63] constructed three machine learning models to describe the limiting potentials for HER, OER, and ORR using easily accessible physicochemical properties. These models were used to predict the limiting potentials of other 260 M@N_x_C_y_ SACs. The reliability of the constructed machine learning models was subsequently verified after screening several optimal catalysts using DFT tests. For OER, Ir@pyridine-N_3_C_1_ and Ir@pyridine-N_2_C_2_ were screened. These catalysts exhibited superior performance compared to the commercial noble metal oxide catalysts RuO_2_ and IrO_2_. Additionally, for HER, Ni@pyridine-N_3_C_1_ demonstrated superior performance compared to Pt-based catalysts. This study highlights the efficiency and reliability of machine learning in identifying high-performing catalysts.

Machine learning is considered a promising approach for the rational design of SACs not only because it accelerates the screening process for high-performance catalysts but also because it establishes a deeper understanding of the relationship between catalytic activity and structural or atomic properties based on catalytic mechanisms [64]. For example, changing the substrate size and dimension can modulate the HER of single-atom catalysts (SACs) [65]. Researchers used the DFT method to calculate ΔG_H*_ for 3d, 4d and 5d transition metal embedded substrates and screened Co-graphene (NG) as the best HER catalyst. Further studies revealed that the change in substrate size and dimension (from graphene to nanographene) changes the electronic structure of the metal, which affects the adsorption of hydrogen on the metal. Ultimately, they used the SISSO model to reveal the relationship between various descriptors and ΔG_H*_, and derived a more accurate general descriptor for describing the catalytic activity of HER:

(2) $$\Delta G_{\mathrm{H*}}=-1.032\times \left(\frac{\varepsilon_{d}}{q}\right)+13.424\times \left(\frac{1}{r_{cov}}\right)+1.726\times \left(\varepsilon_{d}\times EN\right)-0.045\times \left({d_{occ}}^{2}\right)-9.241$$

which consists of four atomic properties, namely the d-state center (*ε_d_*), covalent radius (*r_cov_*), Bader charge (*q*), and number of occupied d states (*d_occ_*). This approach enables rapid screening of HER catalysts with better performance based on further doping of graphene nanographene substrate, and is expected to be applied to other electrocatalytic reactions. Similarly, Liu et al. [59] made great progress in combining machine learning with theoretical calculations and experiments to explore Co single-atom catalysts (Co SACs). They used systematic DFT to calculate the free energies of 27 possible active sites and found that the edge models (Co-2N-A and Co-2N-Z) of Co atoms embedded in graphene-based SACs are more catalytically active than the planar model (Co-4N-P), which is mainly due to the fact that the 3d orbitals of Co of the edge model are localized near the Fermi energy level, whereas the 3d orbitals of Co in the Co 4N-P model, and the d-band center of the edge model is negatively shifted with respect to the planar model. Based on this, they designed and synthesized edge-enriched Co single atoms (E-Co Sas) and employed the machine learning model to elucidate the atomic structure of this Co single atom based on 1000 structural datasets generated from EXAFS simulations. The resulting Co SA local environments are obtained with 65.49%, 13.64%, and 20.86% of Co-4N-P, Co-2N-A, and Co-2N-Z, respectively, which are consistent with the Athena result fit, indicating the accuracy of the machine learning model. Meanwhile, the HER performance of E-Co Sas was significantly improved due to the edge effect compared to CoN_4_. This study reveals that the edge Co atoms are the active sites for HER, and also introduces a new method for machine learning to interpret synchrotron radiation spectra.

**Figure 6:**
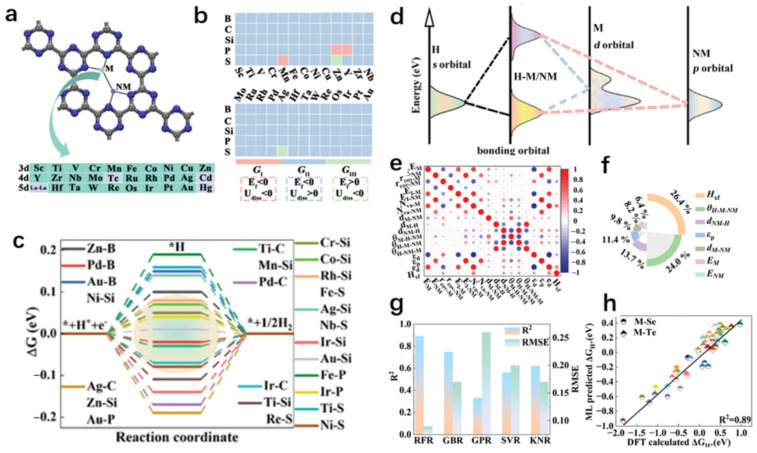
(**a**) The optimized structures for M-NM/g-CN. (**b**) The formation energy and dissolution potential for M-NM/g-CN. (**c**) Schematic diagram of the interaction between H *s* orbitals and M d orbitals (NM p orbitals). (**d**) Gibbs free energy diagram for HER on M-B/g-CN (M = Zn, Pd, Au), M-C/g-CN (M = Ti, Pd, Ag, Ir), M-Si/g-CN (M = Ti, Cr, Mn, Co, Ni, Zn, Rh, Ag, Ir, Au), M-P/g-CN (M = Fe, Ir, Au), and M-S/g-CN (M = Ti, Fe, Ni, Nb, Re). (**e**) Pearson correlation coefficient between the pairwise features. (**f**) The feature importance calculated by RFR for ΔG_H*_. (**g**) The RMSE and the R^2^ on the test set of ΔG_H*_ with RFR, GBR, GPR, SVR, and KNR algorithms. (**h**) The DFT calculated and machine learning predicted ΔG_H*_ for M-Se/g-CN and M-Te/g-CN. Reproduced from Ref. [68] with permission.

Despite these advancements, since the active sites of SACs are single atoms, the loading of active atoms per unit volume or mass of the carrier is relatively low, which limits the wide application of single-atom catalysts to some extent. With the continuous exploration of electrocatalysts, following the SACs, dual-atom catalysts (DACs) have gradually entered into people’s view. Taking bimetallic atom catalysts as an example [66], due to the reduced spatial distance between the atoms, the interactions generated between the metal atoms will result in a catalyst with a unique coupled electronic structure, which will exhibit moderate adsorption and desorption capacity, i.e., better catalytic activity in catalytic reactions. Nowadays, machine learning is more and more frequently applied in the process of exploring catalysts, which likewise contributes to the development of DACs. However, some classical machine learning methods are not applicable in the screening of DACs, mainly due to the flexibility of their active sites, which greatly increases the difficulty of machine learning to extract effective features. In view of this, Namuangruk et al. [67] calculated and screened diatom-doped N_6_-graphene catalysts with high HER catalytic activity. In this work, they adopted the screening criteria of |ΔG_H*_| ≤ 0.05 eV, bandgap ≈ 0 eV, no significant structural distortion at high temperature, negative formation energy, and positive dissolution energy. Additionally, they screened two electrocatalysts, AuCo@N_6_Gr and NiNi@N_6_Gr, with excellent performance from 435 structural combinations of double transition metal atom-doped N_6_-graphene using DFT calculations and a machine learning model. Through specific analyses, it is found that the source of the activity of these two catalysts is the synergistic interaction between bimetallic atoms, which contributes to the rational design of DACs. Similarly, Huang et al. [68] investigated a novel metal–nonmetal-doped g-CN diatomic catalyst (Figure 6). In this study, out of 130 structures consisting of non-metallic elements and 26 metallic elements, 11 high-performance HER catalysts were calculated by DFT high-throughput. By analyzing the electronic structures, these high-performance DACs showed metallic properties because of the contribution of the d orbitals of the metal atoms, which is conducive to electrocatalytic HER. In order to understand the source of their activities, they collected a number of data including the different interatomic bond lengths, bond angles and covalent radii as input features, used the RFR model (R^2^ as high as 0.89 and RMSE as low as 0.09) as a prediction model and successfully predicted two other high-performance electrocatalysts, demonstrating the accuracy of the machine learning model in predicting HER electrocatalysts. This section focuses on summarizing and analyzing the application of machine learning in the rational design and screening of mono/diatomic catalysts and their mechanisms, which further validates the feasibility and reliability of machine learning in the catalyst discovery process.

### 3.3. Two-Dimensional Electrocatalysts

The rise of 2D materials can be traced back to the successful exfoliation of layered graphene by Novoselov [69]. With the continuous and in-depth research on 2D materials, more and more 2D materials have been unearthed, for example, Mxenes, transition metal chalcogenide, carbides, and so on. It is worth mentioning that 2D materials themselves have been among the current HER electrocatalysts development and its related fields by virtue of their special structural and physicochemical properties [70,71]. With a high specific surface area and abundant active sites, 2D materials are also considered as alternative materials to Pt-based noble metal catalysts. However, for the theoretical design aspect of high-performance 2D materials, they still face the problems of long time and high cost of high-throughput calculations, which seriously hinder the discovery of excellent 2D HER electrocatalysts [72]. The emergence of machine learning is expected to break this impasse, and by combining machine learning and high-throughput computation, it can not only reduce the cost of material discovery, but also further clarify its HER catalysis mechanism.

**Figure 7:**
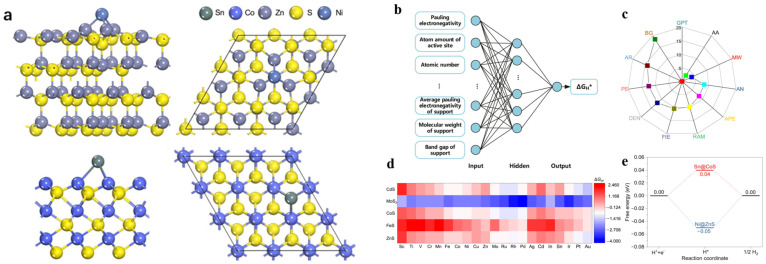
(**a**) Structure models of chalcogenides-supported transition-metal single-atom catalysts. (**b**) BP neural network model. (**c**) Radar chart of the feature importance, squares of different colors represent different input features. (**d**) Heat matrix for the HER catalytic activity of M@TMC catalysts. (**e**) Gibbs free energy diagram of hydrogen evolution reaction of Sn@CoS and Ni@ZnS. Reproduced from Ref. [79] with permission.

Transition metal dichalcogenide (TMD) are very classical two-dimensional materials [73], and many studies have found that TMDs have the advantages of low cost, good stability, and tunable catalytic activity, which are considered to be very promising HER electrocatalysts [74,75]. Machine learning technology is in the ascendant, which brings an opportunity for the further development of TMDs in the field of electrocatalysis. Among many TMDs, MoS_2_, as an excellent HER catalyst [76], has become the focus of extensive researchers’ attention. For example, due to the diversity of nanocluster materials, the traditional ab initio calculation method is difficult to achieve the task of catalyst optimization. Jäger et al. [77] compiled a dataset of 10,000 Δ*E_H_* single-point DFT calculations for MoS_2_ and Au_40_Cu_40_ nanoclusters, which were used as a training set for a machine learning model to analyze in depth the performance of different structural descriptors (SOAP, MBTR, ACSF). It is shown that compared with other structural descriptors, the SOAP descriptor, which represents the local environment of the central atom by Gaussian blurring the positions of neighboring atoms, exhibits high accuracy in predicting the hydrogen adsorption energy. The MAE is as low as 0.13 eV, which provides certain impetus for the exploration of catalysts. In recent years, numerous researchers have devoted themselves to enhancing the HER catalytic performance of materials by atomic doping. However, this doping behavior can influence the electronic structure and properties of the material, potentially altering its chemical activity, stability, and other characteristics. Therefore, further in-depth exploration is still needed. In an earlier work, Hakala et al. [78] used a combination of DFT calculations and machine learning methods to investigate in depth the effect of transition metal (Fe, Co, Ni, and Cu) doping on the structure and hydrogen adsorption properties of MoS_2_. Specifically, they applied DFT to calculate the Gibbs free energy of hydrogen adsorption ΔG_H*_ and the formation energy for assessing the stability of the Mo sites on the MoS_2_ surface when the doped transition metals directly replace them, and constructed a random forest (RF) model by collecting 126 cases consisting of features such as system type, number of valence electrons, number of coordination sites, and adsorption positions as a dataset to perform classification and regression tasks. The results of the study showed that the type of dopant as well as the edge type of MoS_2_ had a crucial influence in predicting the hydrogen adsorption capacity. Tu et al. [79] conducted a similar study, although their research focused on applying machine learning to accelerate the screening of electrocatalysts in a larger compositional space (Figure 7). They constructed a model based on the most stable adsorption structure formed by metal atoms adsorbed on the surface of transition metal chalcogenides (TMCs) (100), probed their HER catalytic performance in depth with DFT, and calculated the hydrogen adsorption free energy change ΔG_H*_. Then, a machine learning model was constructed, and the physicochemical properties of different M@TMCs were used as input features to screen the M@TMC composition space after the model was trained. Through the feature importance analysis of the machine learning model, the band gap of the carrier material was identified as the most important descriptor for the HER performance of the single-atom chalcogenide catalysts, and some intrinsic features of the active center atoms were also found to contribute significantly to the catalytic activity, and two outstanding HER electrocatalysts, Sn@CoS and Ni@ZnS, were successfully screened out.

Unlike catalytic systems consisting of specific metal single-atoms and chalcogenides, Ran et al. [80] explored a broader range of materials by integrating machine learning and DFT computational methods. Their team performed a systematic high-throughput computational screening of all possible two-dimensional TMC (2D-TMD) materials, covering a wide range of structural phases and defect types, including perfect monolayers, transition-metal ionic vacancies (TM-vacancies), and chalcogenide ionic vacancies (X-vacancies), among other structures, the research object is more extensive and comprehensive. They identified 70 metal phase TMD materials as highly promising HER catalysts. Through electronic structure analysis and machine learning modeling, they introduced a novel catalytic activity descriptor:

ΔG_H*_ = 0.093 − (0.195 × *Lef* + 0.205 × *Les*) − 0.15 × *V_tmx_*, (3)

where *LEf* and *Les* represent the local electronegativity of nearest and next-nearest neighbors, while *V_tmx_* denotes the average valence electron number of TM-X. Based on this, a wide variety of 2D with high HER activity were further discovered-TMD materials, such as perfect monolayers of VS_2_ and NiS_2_, TM-vacancy of ZrTe_2_ and PdTe_2_, X-vacancy of MnS_2_, CrSe_2_, TiTe_2_, VSe_2_, and FeS_2_, which have the catalytic activities comparable to Pt (111). This provides a systematic and comprehensive screening process for the search of efficient HER catalysts, which is instructive for new catalyst design and activity optimization.

**Figure 8:**
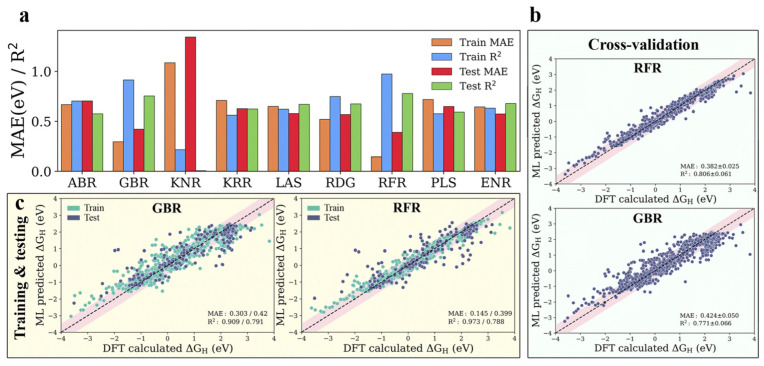

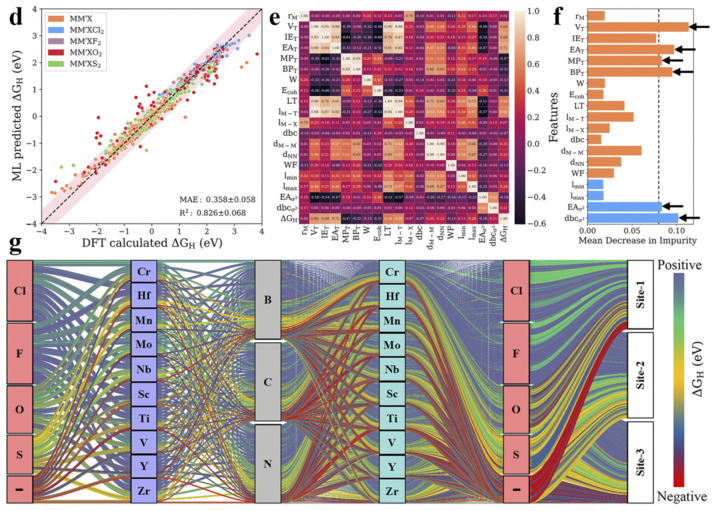
(**a**) Mean absolute error (MAE) and coefficient of determination (R^2^ score) of the ABR, ENR, GBR, KNR, KRR, LAS, PLS, RFR, and RDG algorithms. Parity plots of the best-performing RFR and GBR models (**b**) with and (**c**) without cross-validation using the DFT dataset of hydrogen adsorption Gibbs free energies (ΔG_H*_). (**d**) Parity plot of predicted vs. actual ΔG_H*_ from the GBR model with RFE–HO–LOO in the best cross-validated process. (**e**) Pearson correlation coefficient (PCC) heat map for the reduced set of features after recursive feature elimination (RFE), hyperparameter optimization (HO), and the leave-one-out (LOO) approach. (**f**) Feature importance from the mean decrease in impurity for the GBR model with RFE–HO–LOO, evaluated via 20-fold cross-validation, the black arrows denote the key descriptors highlighting the impact on the hydrogen evolution reaction (HER) activity of MXenes. (**g**) Alluvial diagram for the predicted ΔG_H*_ values of 4500 MM′XT_2_-type MXenes. Reproduced from Ref. [87] with permission.

MXene two-dimensional materials are a general term for a series of recently discovered transition metal carbides, nitrides, and carbon–nitrogen compounds with graphene-like structures [81,82,83,84]. The chemical formula can be expressed as M_n+1_X_n_T_X_ (n = 1−3; M = Sc, Ti, V, Cr, Zr, Nb, Mo, Hf, Ta, etc.; X = C, N; T = O, OH, F, etc.) [85]. Due to the diversity of the structural and compositional species of the two-dimensional MXenes, they have a wide range of applications in the fields of electrochemical energy storage and catalysis. In recent years, 2D MXenes materials have become a new research hotspot for electrocatalytic studies because they can be used as catalysts and catalyst carriers [86]. Many studies have demonstrated the feasibility of 2D MXenes to replace noble platinum-based noble metal catalysts. Machine learning models are extremely versatile and portable, which can also provide guidance and reference for the development of MXenes. For example, in order to rapidly predict the exact Gibbs free energy change (ΔG_H*_) of 4500 MM’XT2-type MXenes, Abraham et al. [87] constructed a multi-step workflow involving data processing, feature engineering, model training, selection, and performance prediction. Using DFT calculations on a random 1125 systems as a material space to assess HER performance and train machine learning models. By comparing and optimizing multiple machine learning models, the gradient boosted regression (GBR) model was identified to be superior in predicting the HER activity of the MXene materials, and the prediction accuracy of the model was further improved and the prediction error was reduced by the techniques of RFE, HO, and LOO, which enabled the model to capture the relationship between the material properties and the structure more accurately. The results show that the number of valence electrons of the termination group, the electron affinity and the variance of the d-band center with respect to the mean value are the determining factors affecting the catalytic activity (Figure 8). This not only helps to understand the mechanism of regulating the HER performance of MXene materials, but also helps to develop more efficient HER catalysts. This study achieves fast and accurate prediction of HER performance of a large number of MXene materials by organically combining DFT calculations with machine learning algorithms, which improves research efficiency. Further advancements were made by Wang et al. [88], who focused on the rational design of 2D MXene-ordered binary alloys (OBAs). Through DFT calculations, they screened 2520 candidate catalysts, and successfully identified 188 MXene OBA catalysts with potentially excellent performances, the |ΔG_H*_| are all less than 0.2 eV. Notably, 110 of these catalysts outperform conventional Pt-based noble metal catalysts in terms of catalytic activity and stability. In order to deeply investigate the intrinsic correlation between the activity of these catalysts and the geometrical and electronic factors, the research team used the AdaBoost integrated learning model to carry out the feature importance calculation. In the process of gradually reducing the feature dimensions, five key features that are closely related to the catalytic performance of HER were identified. Subsequently, these features were comprehensively validated by multiple evaluation tools, confirming their good stability and validity, suggesting that they can be used to reflect the origin of the HER activity of MXene OBA, and that machine learning can open new paths for the design of novel complex catalysts.

Overall, with the increasing number of catalyst types and the growing complexity of catalytic reaction processes, multiple generic descriptors are needed to predict trends in the catalytic activity of 2D MXenes. Data-driven models based on high-throughput screening and machine learning play an important role in discovering generic, accurate, and measurable descriptors.

## 4. Challenges and Perspectives

Hydrogen evolution reaction (HER) occupies a central position in electrochemical hydrogen production, and its efficient conduct is of key significance for achieving sustainable energy development. Currently, catalyst screening and development with the assistance of machine learning have achieved milestones. With the deep combination of high-throughput computing and machine learning techniques, the relationship between microstructure and catalytic performance of HER electrocatalysts has been further explored. Until now, the application of machine learning in catalysis continues to increase, and due to its own powerful data processing and analysis capabilities, machine learning has become an important part of the rational design of HER electrocatalysts, the rapid search for special descriptors, and the comprehensive revelation of reaction mechanisms. However, while we marvel at the achievements of machine learning applied to electrocatalysis, some remaining challenges cannot be ignored, which can be categorized into the following points:

Data collection and quality. In electrocatalysis, high-quality datasets are fundamental for models to accurately predict electrocatalyst performance, thereby facilitating the discovery of outstanding HER electrocatalytic materials. Precise stability, reaction selectivity, and catalytic activity are pivotal in electrocatalyst design. Machine learning can aid this process by analyzing and discerning patterns within large datasets. Nevertheless, the collection and integration of such high-quality datasets pose formidable challenges. In most studies, training data are collected from materials databases, the literature, and high-throughput experiments or calculations. Due to the complexity of the catalysts themselves, the collected data are susceptible to biases, such as limited coverage of the input data in the material space, conflicting data from different sources, and incorrectly chosen descriptors, which can lead to misses of a magnitude. Reliable models can only be developed with precise, high-quality datasets. This, in turn, facilitates the identification of potential novel HER electrocatalysts amidst a multitude of materials, propelling the advancement of the HER electrocatalysis field and the overall progress in catalyst design.

Machine Learning multiscale simulation. In terms of theoretical calculations, although methods such as DFT can provide some guidance, there are still difficulties in simulating real experimental conditions. For example, in the calculation, it is difficult to precisely regulate the temperature, pressure, solution medium, atomic ions, pH, and other factors, which leads to the gap between the theoretical calculation and the actual experiment. Machine Learning multiscale simulations integrate data and models across different scales, but their application is often constrained by research size and complexity. Simulating large-scale catalytic systems under realistic reaction conditions remains computationally expensive and time-intensive, limiting the scalability of these approaches.

Model interpretability. The interpretability of machine learning models pertains to the capacity to comprehend and elucidate the rationale and means underlying model decisions and predictions. In catalyst theoretical design, it facilitates a profound understanding of the electrocatalytic reaction process, enabling an accurate portrayal of the intricate activity–structure relationship, thereby facilitating the prediction of new materials. Nevertheless, the vast quantities of reaction data can convolute the relationships established by machine learning models, potentially giving rise to spurious associations, which undermines model interpretability. Moreover, the electrocatalysis domain interprets reaction mechanisms grounded in a rigorous physical–chemical theoretical framework. Conversely, most machine learning models are data driven for pattern recognition and prediction, lacking inherent physical–chemical logic. When numerous reaction mechanisms remain ambiguous, even if machine learning models can precisely forecast electrocatalytic performance, integrating the prediction outcomes with the unclear mechanisms for interpretation proves arduous. This, to a certain degree, constrains the interpretation of model results.

Lack of common models and standards. The development of HER catalyst screening based on DFT and machine learning is a multidisciplinary field. The data, features, and model applications vary greatly in different studies, which makes it difficult to summarize universal rules, and even the same material system may obtain different results in different studies due to different data and models, which brings troubles to the design and performance evaluation of HER electrocatalysts. In future research, enhancing model generality and establishing model evaluation standards can help to achieve interdisciplinary and cross-field integration and application, and improve the synergy efficiency of research work.

Currently, HER faces many challenges, and solving these challenges will enable the combination of high-throughput computing and machine learning to play a greater potential in catalyst design and provide more convenient tools for the in-depth development of the catalysis field. Meanwhile, with the rapid development of technologies such as machine learning and big data, the development of catalysts will move towards intelligence and integration, which will greatly improve design efficiency and quality and deepen the understanding of molecular interactions. This will also provide more accurate calculation results and more comprehensive theoretical guidance for experimental work, accelerate the discovery of new and efficient catalysts, and thus promote the development of hydrogen energy technology.

## Figures and Tables

**Table 1:** Advantages and disadvantages of classical machine learning models and their scope of application.

Model	Advantages	Disadvantages	Scope of Application
MLR	Simple and easy to understand High computational efficiency Relatively strong interpretability	Can only handle linear relationships	Approximately linearly correlated data
		Poor fitting effect for complex nonlinear problems	
SVM	Good performance on small sample data Strong generalization ability Capable of handling nonlinear problems	Complicated selection of kernel functions	Small sample data Nonlinear data High-dimensional data
		Parameter tuning is difficult	
		The calculation is complex.	
DT	Intuitive and interpretable Capable of handling classification and numerical data Insensitive to outliers Easy to explain and understand	Prone to overfitting Poor model stability Slow prediction speed	Data with clear feature attributes
			Data that can be classified or used for continuous value prediction
			Imbalanced data
RF	High accuracy	High computational cost	High-dimensional data Imbalanced data
	Not prone to overfitting	Slow prediction speed	
	Capable of handling complex data	Poor interpretability	
XGB	High speed in training	Prone to overfitting Hard to adjust parameters.	High quality data Large scale data
	High error-tolerant rate		
	Easy to explain and understand		
